# An evaluation of performance measures for arterial brain vessel segmentation

**DOI:** 10.1186/s12880-021-00644-x

**Published:** 2021-07-16

**Authors:** Orhun Utku Aydin, Abdel Aziz Taha, Adam Hilbert, Ahmed A. Khalil, Ivana Galinovic, Jochen B. Fiebach, Dietmar Frey, Vince Istvan Madai

**Affiliations:** 1 CLAIM - Charité Lab for Artificial Intelligence in Medicine, Charité Universitätsmedizin Berlin, Berlin, Germany; 2 Research Studio Data Science, Research Studios Austria, Salzburg, Austria; 3 Centre for Stroke Research Berlin, Charité Universitätsmedizin Berlin, Berlin, Germany; 4 Department of Neurology, Max Planck Institute for Human Cognitive and Brain Sciences, Leipzig, Germany; 5 Mind, Brain, Body Institute, Berlin School of Mind and Brain, Humboldt-Universität Berlin, Berlin, Germany; 6 QUEST Center for Transforming Biomedical Research, Berlin Institute of Health (BIH), Charité - Universitätsmedizin Berlin, Berlin, Germany; 7 Faculty of Computing, Engineering and the Built Environment, School of Computing and Digital Technology, Birmingham City University, Birmingham, UK

**Keywords:** Cerebral vessel segmentation, Segmentation measures, Cerebral arteries, Average Hausdorff distance, Dice, Segmentation, Ranking, Image processing (computer-assisted)

## Abstract

**Background:**

Arterial brain vessel segmentation allows utilising clinically relevant information contained within the cerebral vascular tree. Currently, however, no standardised performance measure is available to evaluate the quality of cerebral vessel segmentations. Thus, we developed a performance measure selection framework based on manual visual scoring of simulated segmentation variations to find the most suitable measure for cerebral vessel segmentation.

**Methods:**

To simulate segmentation variations, we manually created non-overlapping segmentation errors common in magnetic resonance angiography cerebral vessel segmentation. In 10 patients, we generated a set of approximately 300 simulated segmentation variations for each ground truth image. Each segmentation was visually scored based on a predefined scoring system and segmentations were ranked based on 22 performance measures common in the literature. The correlation of visual scores with performance measure rankings was calculated using the Spearman correlation coefficient.

**Results:**

The distance-based performance measures balanced average Hausdorff distance (rank = 1) and average Hausdorff distance (rank = 2) provided the segmentation rankings with the highest average correlation with manual rankings. They were followed by overlap-based measures such as Dice coefficient (rank = 7), a standard performance measure in medical image segmentation.

**Conclusions:**

Average Hausdorff distance-based measures should be used as a standard performance measure in evaluating cerebral vessel segmentation quality. They can identify more relevant segmentation errors, especially in high-quality segmentations. Our findings have the potential to accelerate the validation and development of novel vessel segmentation approaches.

**Supplementary Information:**

The online version contains supplementary material available at 10.1186/s12880-021-00644-x.

## Introduction

Stroke is a leading cause of mortality and disability, affecting 15 million people worldwide [1]. As a cerebrovascular disease, it is characterised by arterial brain vessel changes, e.g. narrowing and occlusion. Thus, the status of the cerebral arteries is routinely utilised in the clinical setting for the understanding, treatment and prevention of stroke [2]. For example, quantified parameters such as arterial diameters can serve as biomarkers for foreseeing future strokes [3]. Additionally, the incompleteness of intracranial vessel structures, such as the circle of Willis was associated with a higher risk of anterior circulation stroke [4]. In addition, other diseases such as vessel inflammations or aneurysms can lead to changes in the vasculature. Therefore, accurate visualisation and quantification of the status of the arterial vessel tree are of high clinical relevance.

Recently, advances in deep neural network architectures, a particular type of artificial intelligence (AI), made fully automated and clinically applicable cerebral vessel segmentation approaches feasible [5–7]. Once deployed, these methods do not rely on human intervention and can provide high-quality binary segmentations of the arterial vessels in less than a minute [5]. However, a severe obstacle to developing and validating improved vessel segmentation approaches is accurate segmentation performance assessment. In other words, how do we know which model provides better segmentations?

Usually, the performance assessment of a given segmentation result encompasses a qualitative and quantitative analysis. Qualitative analysis is done visually; however, its inter-rater variability, susceptibility to human error and time-consuming nature limit its broader use [8, 9]. The quantitative analysis comprises the comparison of a given segmentation to a reference image via a computed measure. The reference image—also called the ground truth—is usually a manual segmentation performed by at least one human expert. The comparison is performed via specific performance measures. Taha et al. provide an extensive overview of the existing measures [10]. In brief, many measures exist, and they can be divided into distinct families: overlap based, volume based, pair counting based, information theoretic based, probabilistic based and spatial distance based measures [10]. Each type of performance measure is sensitive to different types of errors present in a segmentation. Also, each measure has other biases depending on the characteristics of the segmented structures. Therefore, to assess segmentation performance measures should be selected that are the best fit for each given segmentation task.

For arterial brain vessel segmentation, specifically, various performance measures are in widespread use for evaluation of vessel segmentation quality [11].

The most commonly used measure is the Dice coefficient [12, 13]. It is popular because it is easily interpretable and allows comparisons with other studies [14]. Less often, other performance measures such as the average Hausdorff distance [15], the area under the receiver operating characteristic curve [16], sensitivity [17, 18], specificity [18], or accuracy [16–18] are used.

Importantly, however, there is no scientific evidence supporting that the Dice coefficient—or any other measure—in arterial brain vessel segmentation is the best choice. While theoretical considerations argue heavily in favour of distance-based measures [10], an empirical assessment to corroborate or refute these theoretical assumptions lacks to date.

Therefore, in the present work, we aimed to fill this scientific gap. To find the most suitable performance measures for cerebral vessel segmentation, we first simulated segmentation variations containing various manually created errors. We then visually scored these segmentations using a predefined scoring system. Finally, we correlated these visual scores with the segmentation rankings provided by 22 different performance measures to find the most suitable measure.

## Methods

### Data

Time-of-flight MR-Angiography (TOF MRA) images of 10 patients from the 1000Plus study were randomly selected. The 1000plus study included patients with the clinical diagnosis of an acute cerebrovascular event within the last 24 h. For our analysis, the only inclusion criterion was a complete Circle of Willis without any occlusion in its vessel segments. The reason for this inclusion criterion was that patients with occlusions in the arteries of the Circle of Willis would not allow the creation of errors in these arteries. The 1000Plus study was carried out with approval from the local Ethics Committee of the Charité University Hospital Berlin (EA4/026/08). Details about the study have been previously published [19].

### Imaging parameters

Time-of-flight MR-Angiography (TOF MRA) was performed on a 3T MRI scanner (Tim Trio; Siemens AG, Erlangen, Germany) with the following parameters: Voxel size = (0.53 × 0.53 × 0.65) mm3; Matrix: 364 × 268; Averages: 1; TR/TE = 22 ms/3.86 ms; Gap: − 7.2; FOV: 200 mm; Duration: 3:50 min; Flip angle = 18 degrees.

### Ground truth creation

**Fig. 1:**
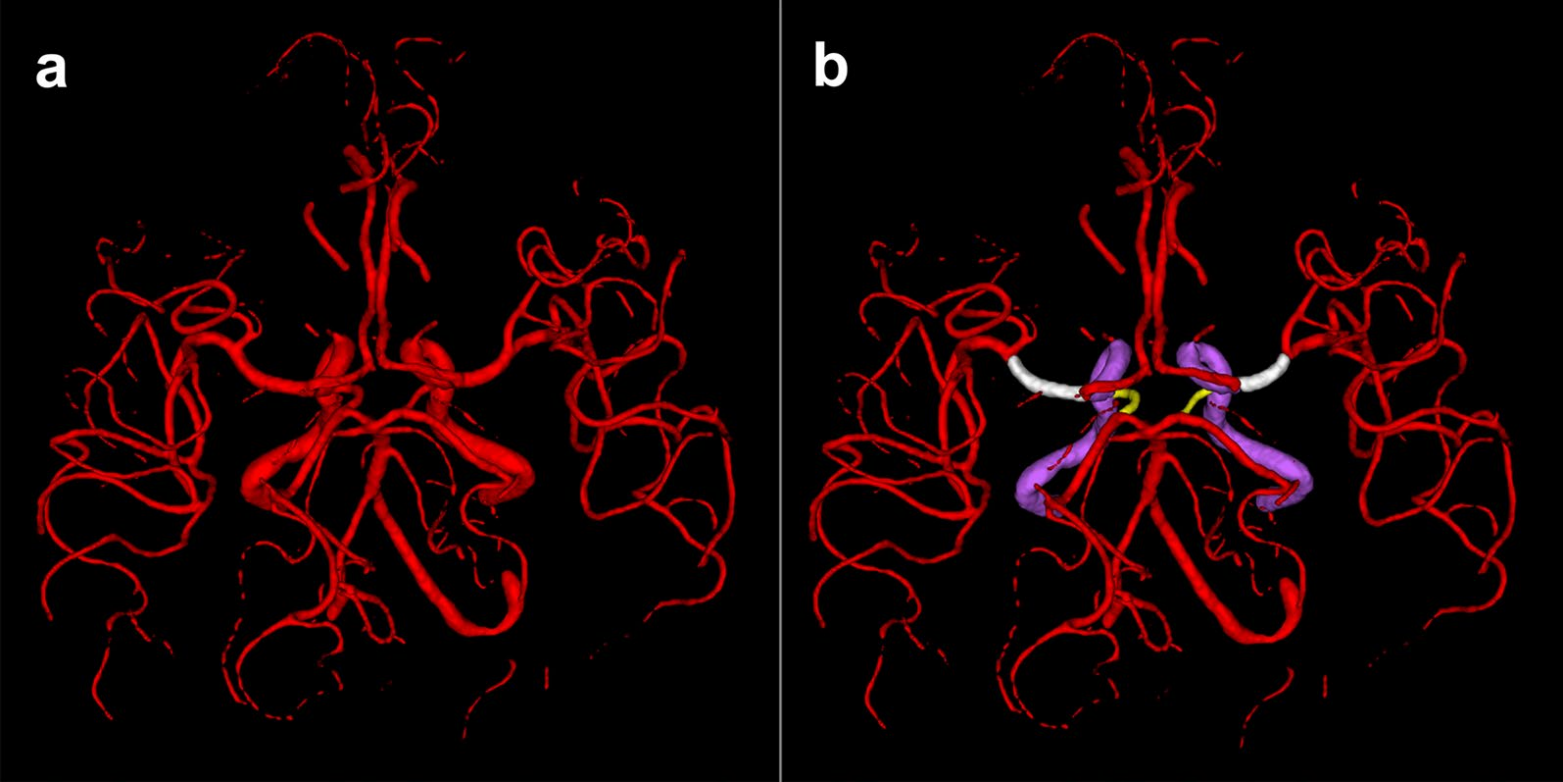
Binary ground truth (**a**) and voxel-wise annotated ground truth (**b**). White: M1 segment of the middle cerebral artery, Yellow: Posterior communicating artery, Purple: Internal carotid artery, Red: Other arteries and artery segments classified as small vessels

To create a ground truth image of the cerebral arterial vessels, the 3D TOF MRA was pre-segmented using a U-net deep learning framework [8] and manually corrected by OUA (4 years experience in stroke imaging) using ITK-Snap [20]. The results were checked by VIM (11 years experience in stroke imaging). The resulting binary ground truth was manually annotated voxel-wise into following arteries and their corresponding segments: internal carotid artery (ICA), the sphenoidal segment of the middle cerebral artery (M1), posterior communicating artery (Pcom). All other segmented arteries were classified as small vessels (Fig. 1).

### Error creation

**Fig. 2:**
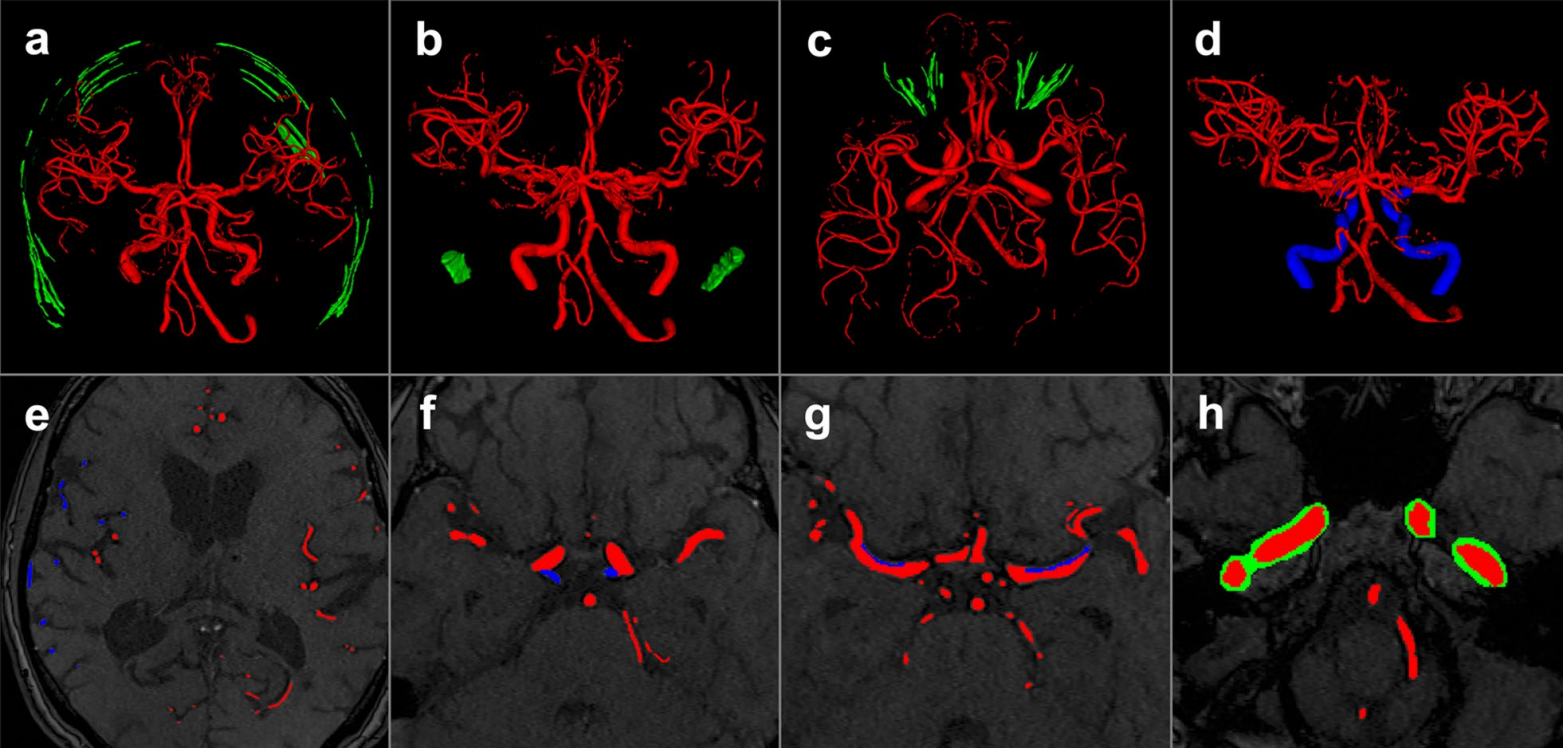
Examples of manually created errors of various intensity levels that were introduced to the ground truth. Examples of false-positive segmentation of structures in green (**a**–**c**): **a** moderate skull error, **b** severe sigmoid sinus error, **c** severe orbit error. Examples of false-negative segmentation of vessels in blue (**d**–**f**): **d** omission of internal carotid artery, **e** severe small vessel error, **f** omission of the posterior communicating arteries. Radius manipulation of segments (**g**, **h**): **g** subtle boundary error of the M1 segment of the middle cerebral artery, **h** severe boundary error of the internal carotid artery. Red: True positive voxels, Green: False-positive voxels, Blue: False-negative voxels

To explore the properties of performance measures for quality assessment of cerebral vessel segmentations systematically, a framework to simulate segmentation variations was developed. To simulate segmentation variations for ranking, a set of 48 non-overlapping segmentation errors commonly encountered in a vessel segmentation task were manually created. In this context, an error means that the ground truth was manipulated manually by introducing false negative or false positive voxels. The created errors are selected based on the experience of our group developing and optimising vessel segmentation algorithms. These errors were regularly encountered in segmentations produced by state of the art deep learning models [5, 8] and also other traditional methods like region growing or graph cut algorithms [8]. Additionally, these errors are also encountered in the literature [21–25]. The errors included, for example, boundary errors of various vessel segments, false positively labelled anatomical vessel and non-vessel structures such as the sagittal sinus, middle meningeal artery, fat and muscle tissue and omitted parts of the vessel tree. Three different intensity levels (subtle, moderate, severe) of errors were generated where possible. Error groups and individual errors created in the framework are listed in Table 1. Example illustrations of errors belonging to different error groups can be found in Fig. 2 and visualisations of all errors can be found in the Additional File 1.

**Table 1:** Manually created errors for simulation of segmentation variations

Error type	False-positive errors (added voxels)	False-negative errors (missing voxels)	Boundary precision errors (radius manipulation of segments)
Number of errors (total = 48)	8 distinct errors with 3 intensity levels (24 errors in total)	4 distinct errors of which one has 3 intensity levels (6 errors in total)	6 distinct errors with 3 intensity levels (18 errors in total)
Name of errors	Superior sagittal sinus (1,2,3)	Small vessels (1,2,3)	Pcom under (1,2,3)
	Middle meningeal artery (1,2,3)	Pcom missing	Pcom over (1,2,3)
	Meninges (1,2,3)	ICA missing	ICA under (1,2,3)
	Sigmoid Sinus (1,2,3)	M1 missing	ICA over (1,2,3)
	Orbit (1,2,3)		M1 under (1,2,3)
	Skull (1,2,3)		M1 over (1,2,3)
	Merge/separation (1,2,3)		
	Random voxels (1,2,3)		

All created errors (n = 48) in the framework are listed and divided into three groups. In parentheses, the error intensity levels for each error are specified (1:subtle 2:moderate 3:severe). Abbreviations: ICA: Internal Carotid Artery, Pcom: Posterior communicating artery M1: Sphenoidal segment of the middle cerebral artery, Random voxels: Selection of random voxels (Subtle: 1%, Moderate: 2%, Severe: 3% of all ground truth voxels) from the original TOF MRA and addition to the ground truth image. Merge/Separation: merging vessels close to each other (A2 segments or M3-M4 segments). Radius manipulation of segments are also false-positive and false-negative errors but are given as a separate category. Detailed illustrations and descriptions of errors can be found in the Additional File 1

### Simulation of segmentation variations

**Fig. 3:**
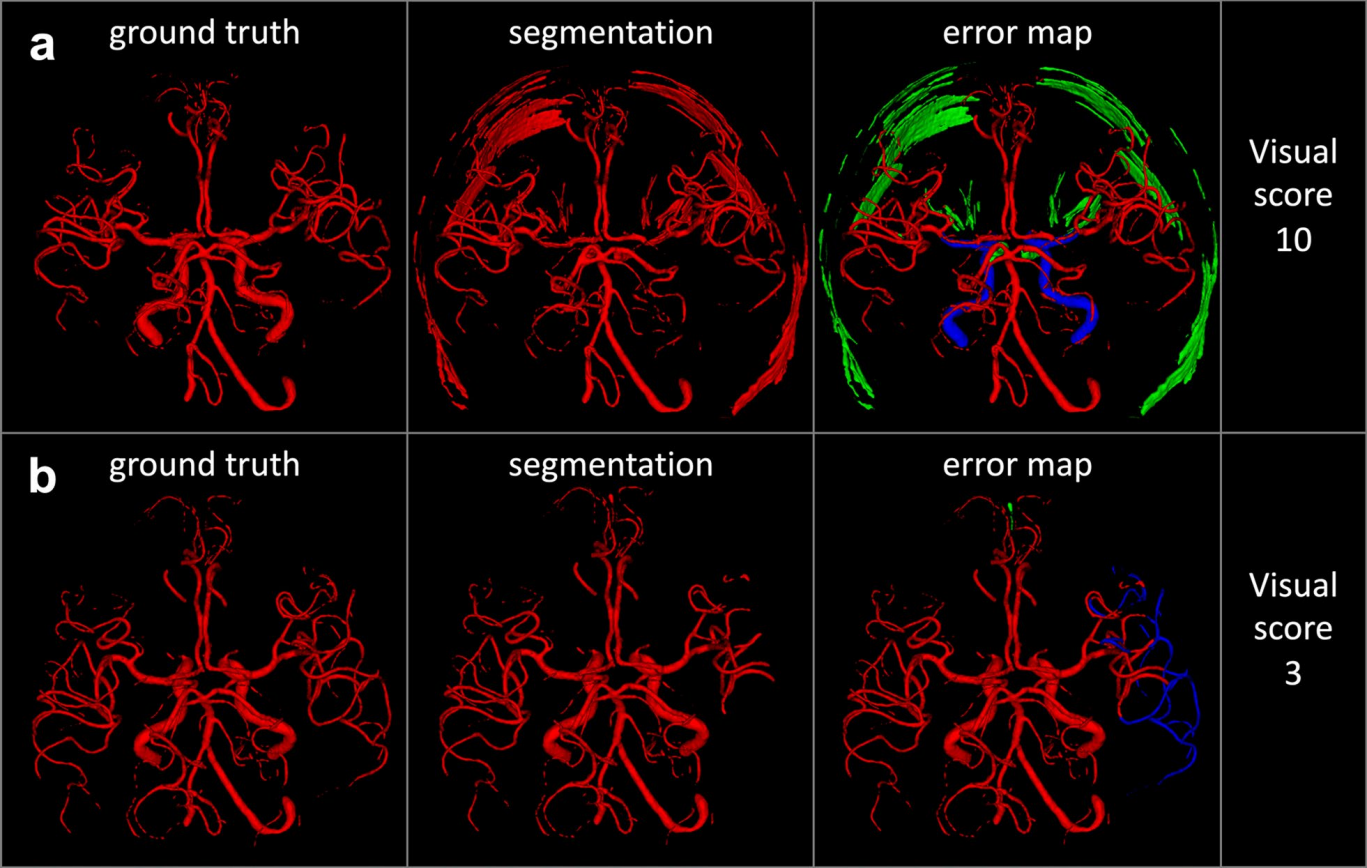
Example simulated segmentation variations containing error combinations and corresponding visual scores. **a** This simulated segmentation variation contains 6 errors: severe orbit error, severe skull error, subtle merge/separation error, omission of the internal carotid artery, severe boundary error of the M1 segment of the middle cerebral artery and posterior communicating artery. Due to the high number and severity of errors, a visual score of 10 is assigned to this segmentation, indicating low quality. **b** This simulated segmentation variation contains 2 errors: a severe omission error of the small vessels and a subtle false-positive segmentation of parts of the superior sagittal sinus. This segmentation gets a visual score of 3, corresponding to moderate quality. Please see Table 2 for the subjective scoring system and Table 1 for a detailed description of errors. Red: True positive voxels, Green: False-positive voxels, Blue: False-negative voxels

In real-world segmentation of cerebral arteries, errors regularly occur in combinations. The simulation framework, therefore, allows combinations of errors. Example error combinations are shown in Fig. 3. To ensure an equal representation of errors in the created sets, the simulated segmentation variations were generated by selecting errors randomly from an error pool of 48 errors with each error having an equal probability to be selected. However, some errors are mutually exclusive because of overlapping voxels that manipulate the same segment or location within the arterial vessel tree volume. This would lead to an unbalanced representation of errors in the analysis where some errors would be unintentionally found more frequently. This unwanted effect was compensated for by defining boundary conditions for segmentation sets**:** First, for each patient, a set was supposed to contain 295 to 305 simulated segmentation variations. Second, in each set, the simulated segmentation variations were supposed to contain a minimum of 2 errors and a maximum of 7 errors per segmentation leading to a total of 6 segmentation groups per set. Third, we also balanced how often these error groups appeared per patient set. Each group was allowed to appear 45–60 times. Finally, to prevent an over-representation of specific errors, each manually created error occurred a minimum of 25 and a maximum of 30 times in total in each set.

### Software environment

Our framework was written in the Python programming language. For the introduction of errors to the ground truth, we used the Python library NiBabel to add or subtract images in *NIfTI* data format. Random combinations were achieved with the *combinations* function from the *itertools* module in Python. Error combinations that were not allowed are specified within the code. The ranking was performed using the *min* method of the *rank* function in Pandas library in Python. The code is available under the following GitHub repository: https://github.com/prediction2020/arterial_vessel_measures.

### Visual scoring

Each simulated segmentation variation was visually scored based on a newly designed predefined scoring system with scores ranging from 1 to 10. Higher visual scores denote higher severity of errors in the simulated segmentation variations and lower segmentation quality. For example, a score of 10 was assigned to segmentations containing multiple severe errors, whereas a score of 1 was assigned to segmentations with subtle errors not affecting segmentation quality. The visual scoring system is described in Table 2. The scoring was performed by OUA with 4 years of experience in cerebral vessel segmentation. A total of 2984 segmentations were scored with approximately 300 from each of the 10 patients. Example visualisations of two simulated segmentation variations with their corresponding visual scores can be found in Fig. 3.

**Table 2:** Criteria of the predefined visual scoring system for simulated segmentation variations

Score	Combined error severity	Segmentation quality	Scoring criteria
1	Low	High	• Minor errors with a typically low number of false positive or false negative voxels with minimal deterioration of segmentation quality **and/or** • Minor to moderate boundary errors
2			• False-positive labelling of a low number of voxels not associated with any anatomical structure **and/or** • False-positive labelling of parts of an anatomical structure **and/or** • Parts or arterial segments of the arterial tree are represented without major errors **and/or** • Moderate to severe boundary errors
3	Moderate	Moderate	• False-positive labelling of at least one defined anatomical structure **and/or** • Parts or arterial segments of the arterial tree are missing **and/or** • Severe boundary errors
4			
5			• False-positive labelling of anatomical structures in multiple locations and slices **and/or** • Major parts or arterial segments of the arterial tree are missing
6			
7	High	Low	• False-positive labelling of anatomical structures in multiple locations and slices significantly compromising segmentation quality **and/or** • Major parts or multiple arterial segments of the arterial tree are missing
8			
9			• No/failed discrimination between vessels and other anatomical structures **and/or** • Major parts or multiple major artery segments of the arterial tree are missing
10			

An error severity score was assigned to each simulated segmentation variation based on visual assessment. Higher scores indicate higher combined severity of errors in the segmentation and therefore lower quality of the segmentation. For example, simulated segmentation variations with a score of 7 to 10 are considered low quality and receive a high severity score. Due to the “and/or” criterion one point from each category is enough to assign a score. In higher error severity scores criteria from lower scores can also be fulfilled. For instance, a segmentation with a score of 9 can contain a severe boundary error but this criterion is not listed again under the criteria for score 9 since it is mentioned previously within the criteria of score 3

A senior rater (VIM) validated a random subset of 50 simulated segmentations by performing an independent visual scoring. We assessed differences between the scorings by VIM and OUA by calculating the median score deviation, the interquartile range, the exact score overlap, and the percentage of cases where the raters chose the same subcategory of the scoring scheme (i.e. low/moderate/high quality).

### Performance measures analysis

The simulated segmentation variations were compared against the ground truth using the EvaluateSegmentation software tool [10]. EvaluateSegmentation is an evaluation framework for medical image segmentation comprising implementation of various performance measures from the literature to assess segmentation quality. In addition to the average Hausdorff distance, the tool also included an improved version of the average Hausdorff distance called the balanced average Hausdorff Distance that was introduced recently [26]. The 95th quantile of the Hausdorff distance was utilised to handle outliers [27]. All distance-based measures were calculated in voxels. Complementary to the available measures in the evaluation framework, we added further performance measures used in the literature, namely Conformity and Sensibility [28]. In total, we thus analysed 22 performance measures. These measures belonged to the following categories: Overlap based, volume based, pair counting based, information theoretic based, probabilistic based, and spatial distance based. Details and calculations of the performance measures implemented can be found in the publication of Taha et al. [10] and Table 3.

**Table 3:** Overview of performance measures analysed in this study

Performance Measure	Abbreviation	Category
Dice	DICE	1
Jaccard Index	JAC	1
Sensitivity	SNS	1
Specificity	SP	1
Sensibility	SB	1
Global consistency error	GCE	1
Conformity	CNF	1
Accuracy	ACC	1
Precision	PRC	1
Volumetric similarity	VS	2
Rand Index	RI	3
Adjusted Rand Index	ARI	3
Mutual information	MI	4
Variation of information	VOI	4
Interclass correlation	ICC	5
Probabilistic distance	PBD	5
Cohen's kappa	KAP	5
Area under ROC Curve	AUC	5
Hausdorff distance (95th quantile)	HD95	6
balanced average Hausdorff distance	bAHD	6
average Hausdorff distance	AHD	6
Mahalanobis Distance	MHD	6

The symbols in the “abbreviation” column are used to denote the performance measures throughout the manuscript. The column “category” assigns each metric to one of the following performance measure families: (1) Overlap based, (2) Volume based, (3) Pair counting based, (4) Information theoretic based, (5) Probabilistic based, and (6) Spatial distance based

Simulated segmentation variations were ranked by ordering segmentations according to their performance measure values. Each performance measure provided a score for each analysed simulated segmentation variation denoting how similar or different segmentations were compared with the ground truth. The segmentation with the highest similarity with the ground truth ranked first, and the one with the lowest similarity ranked last within that segmentation set. Each performance measure assigns different scores to segmentations thus producing different rankings. Therefore, one can compare performance measures by comparing the segmentation rankings produced by them. We produced and analysed rankings of segmentations by all 22 performance measures.

Then, we aimed to select the most suitable performance measure by measuring the correlation of the performance measures rankings with the ranking assigned by the visual scores. This is a modified version of the method described by Taha et al. [9]. The visual scores can be thought of as manually assigned ranks to segmentations. The Spearman correlation coefficient was used to measure correlation for the simulated segmentation variation set of each patient individually yielding 10 correlation coefficients. For each measure, the median correlation coefficient was reported. Performance measures were ranked from the highest correlation to the lowest (Table 4). Ranking results of performance measures are reported in standard competition ranking.

**Table 4:** Median spearman correlation coefficients of visual scores and performance measure rankings

Overall correlation results (Visual scores 1–10)			Correlation results of good quality simulated segmentation variations (Visual scores 1–5)			Correlation results of bad quality simulated segmentation variations (Visual scores 6–10)		
Rank	Performance Measure	rho	Rank	Performance measure	rho	Rank	Performance measure	rho
1	bAHD	0.956	1	bAHD	0.817	1	bAHD	0.894
2	AHD	0.950	2	AHD	0.800	2	AHD	0.880
3	RI	0.936	3	VOI	0.758	3	VOI	0.872
3	ACC	0.936	4	GCE	0.757	3	GCE	0.872
3	GCE	0.936	5	ACC	0.754	5	ARI	0.865
3	VOI	0.936	5	RI	0.754	5	ACC	0.865
7	ARI	0.932	7	KAP	0.742	5	RI	0.865
7	KAP	0.932	7	ARI	0.742	8	KAP	0.864
7	PBD	0.932	7	PBD	0.742	8	PBD	0.864
7	DICE	0.932	7	DICE	0.742	8	DICE	0.864
7	ICC	0.932	7	ICC	0.742	8	JAC	0.864
7	JAC	0.932	7	JAC	0.742	8	CNF	0.864
7	CNF	0.932	7	CNF	0.742	8	ICC	0.864
14	PRC	0.858	14	PRC	0.709	14	PRC	0.802
15	SP	0.820	15	SP	0.683	15	SP	0.714
15	SB	0.820	15	SB	0.683	15	SB	0.714
17	MI	0.755	17	MHD	0.621	17	VS	0.532
18	MHD	0.728	18	MI	0.595	18	MI	0.426
19	VS	0.722	19	VS	0.555	19	MHD	0.343
20	HD95	0.418	20	HD95	0.359	20	HD95	0.259
21	AUC	0.378	21	AUC	0.271	21	AUC	0.142
22	SNS	0.314	22	SNS	0.212	22	SNS	0.104

**Fig. 4:**
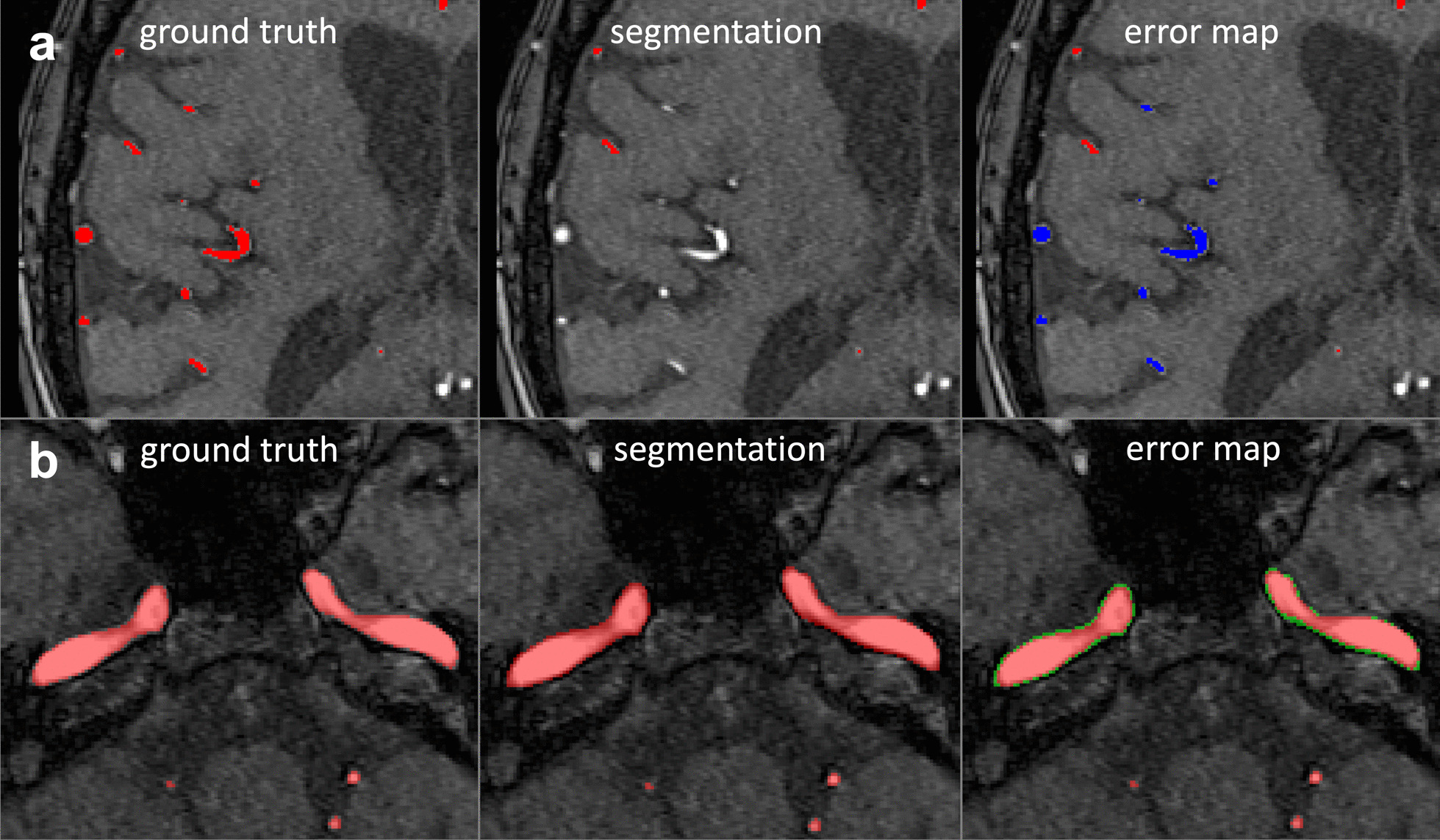
Comparison of bAHD and Dice values for two examples of manually created errors. **a** A severe omission of small vessels is shown in the subpanel **a**. This error received a Dice score of 0.960 and a bAHD score of 0.65. **b** In subpanel **b**, a subtle boundary error of the internal carotid artery is shown**.** This error received a similar Dice value of 0.963, however, it received a lower bAHD value of 0.039. Please note that distance based measures assign lower values to better segmentations. bAHD is sensitive to the error in subpanel a and penalises the omission of small vessels because it considers voxel localisation. In contrast, Dice, which measures only the overlap, cannot distinguish between the two errors. Red: True positive voxels, Green: False-positive voxels, Blue: False-negative voxels

The median correlation of visual scores and performance measure rankings are given for the 10 patients. Together with the overall results analysed over all visual scores ranging from 1–10 (column 1), the results of 2 additional subsets based on the lower (1–5) and upper (6–10) range of the visual scores are reported (columns 2 and 3, respectively). The performance measure names are sorted based on their Spearman correlation coefficient from highest to lowest. Average Hausdorff distance and balanced average Hausdorff distance perform best in the overall analysis as well as in the good and bad quality subsets. In the good quality subset, the difference between average distance-based measures (bAHD and AHD) and overlap based measures is more prominent than in the bad quality subset. This can be interpreted by the relative inability of overlap based measures to distinguish between certain types of errors as shown in Fig. 4. This inability becomes more evident in segmentations of good quality. The group of overlap based measures (Dice, Jaccard, Conformity) have the same correlation in all analyses. Please note that the overall correlation results are inherently higher than the results of the two subsets because the underlying score range of all segmentations (1–10) is wider than the score ranges of the subsets (1–5 and 6–10 respectively). rho: median Spearman correlation coefficient

### Subgroup analysis

We repeated the above-described analysis steps in two subsets to analyze the difference in performance measure rankings based on segmentation quality. The first subset consisted of segmentations of high and moderate quality (visual scores from 1 to 5) and the second subset consisted of segmentations of moderate to low quality (visual scores from 6 to 10).

### Sensitivity analysis of performance measures

In a second subanalysis, we assessed the sensitivity of the applied performance measures to the created errors. An ideal performance measure should have a wide score range and reflect the difference in quality of the assessed segmentations in its values. The extent of the score range shows the sensitivity of a performance measure to the created errors and can be measured by the index of dispersion (IoD). The index of dispersion is calculated by dividing the variance by the mean. We calculated the index of dispersion for each performance measure over the values they assigned to all 2984 simulated segmentation variations.

In addition, it can be challenging to compare the absolute values of performance measures [29]. It becomes easier to compare values when for each visual score the corresponding performance measure values are provided. Therefore, across all patients, for each visual score from 1 to 10, we calculated the median values of performance measures of all simulated segmentation variations receiving this score.

## Results

In our analysis of 2984 simulated segmentation variations, average distance based performance measures performed best. Balanced average Hausdorff distance (rank 1) and average Hausdorff distance (rank 2) provided the segmentation rankings with the highest median correlation with visual scores. Overlap based measures such as Dice, Jaccard, Conformity performed worse (rank 7). Other popular measures such as Volumetric similarity (rank 19) and 95% Hausdorff distance (rank 20) showed considerably lower correlations than the aforementioned performance measures. In 8 out of the 10 tested patients, an average distance based performance measure, either the bAHD or the classic AHD, led the rankings (see Additional File 2). The rankings of all performance measures can be found in Table 4.

In the subgroup analysis, bAHD and AHD were also the best performing measures for both good and bad quality groups. We provide, as an example, two errors in Fig. 4 with their corresponding Dice and bAHD values.

In our second subanalysis, performance measures exhibited different score ranges as evidenced by the index of dispersion (Table 5). The highest IoDs, indicating a beneficial wide spread, were found for the three Hausdorff distance based measures. Generally, the IoDs exhibited large differences, e.g. Conformity (IoD of 0.336) vs. accuracy (IoD of > 0.000002). The balanced average Hausdorff distance had at all times higher IoD values compared with its counterpart, the traditional average Hausdorff distance.

**Table 5:** Index of dispersion and median performance measure values of performance measures

	Median value corresponding to visual score										
PM	IoD	1	2	3	4	5	6	7	8	9	10
HD95	9.191	20.395	43.578	57.892	59.363	61.011	66.332	66.393	67.971	71.365	77.772
bAHD	5.925	0.086	0.332	0.905	1.353	2.309	3.275	4.309	6.408	8.626	13.212
AHD	2.537	0.082	0.318	0.843	1.197	1.983	2.722	3.648	4.766	5.769	7.967
CNF	0.337	0.963	0.925	0.848	0.759	0.701	0.615	0.477	0.353	0.257	-0.145
SB	0.163	0.976	0.957	0.900	0.847	0.784	0.738	0.654	0.524	0.399	0.158
MHD	0.103	0.038	0.077	0.135	0.204	0.207	0.233	0.284	0.309	0.340	0.404
JAC	0.037	0.964	0.931	0.868	0.806	0.770	0.722	0.657	0.607	0.574	0.466
PRC	0.034	0.976	0.956	0.908	0.862	0.816	0.777	0.726	0.660	0.610	0.515
ARI	0.017	0.982	0.964	0.929	0.891	0.868	0.836	0.790	0.751	0.726	0.631
KAP	0.017	0.982	0.964	0.929	0.892	0.869	0.838	0.792	0.754	0.728	0.634
ICC	0.016	0.982	0.964	0.930	0.892	0.870	0.838	0.793	0.756	0.729	0.636
DICE	0.016	0.982	0.964	0.930	0.892	0.870	0.838	0.793	0.756	0.729	0.636
SNS	0.013	0.994	0.989	0.980	0.978	0.970	0.955	0.951	0.935	0.966	0.898
VS	0.011	0.986	0.974	0.956	0.924	0.908	0.888	0.848	0.827	0.780	0.734
VOI	0.010	0.004	0.006	0.011	0.017	0.020	0.024	0.028	0.034	0.039	0.052
AUC	0.003	0.997	0.994	0.989	0.988	0.985	0.977	0.975	0.966	0.981	0.947
GCE	0.002	0	0.001	0.001	0.002	0.002	0.003	0.004	0.004	0.005	0.007
MI	0.001	0.041	0.039	0.037	0.036	0.035	0.034	0.032	0.031	0.031	0.027
PBD	0.001	0	0	0	0	0.001	0.001	0.001	0.001	0.001	0.002
RI	0.000008	1	0.999	0.999	0.998	0.997	0.997	0.996	0.995	0.994	0.991
ACC	0.000002	1	1	0.999	0.999	0.999	0.998	0.998	0.997	0.997	0.996
SP	0.000002	1	1	1	0.999	0.999	0.999	0.998	0.998	0.997	0.996

Performance measures (PM) are sorted from highest index of dispersion (IoD) to the lowest. Median performance measure values corresponding to the different segmentation qualities are provided in the additional columns. Performance measures exhibit different value ranges (Please see Fig. 4 for two errors with their corresponding Dice and balanced average Hausdorff distance values). Some performance measures, such as accuracy and rand index, give close values to segmentations receiving different visual scores. For example, the Rand index has the same median correlation coefficient value of 0.997 for visual scores 5 and 6. In contrast, the adjusted Rand index can differentiate between two visual qualities and has the median values 0.868 and 0.836 for visual scores 5 and 6, respectively. This explains the higher IoD for the adjusted Rand index. Performance measures like Conformity, Sensibility and balanced average Hausdorff distance provide higher IoD values than their theoretical counterparts Dice, Specificity and average Hausdorff distance respectively

The validation analysis of visual scores resulted in a median score deviation of 1 (interquartile range 2), the exact score overlap was 26%, and the raters chose the same subcategory of the scoring scheme (i.e. low/moderate/high) in 78% of cases.

## Discussion

In the present work, we developed a performance measure selection framework based on visual scoring to find the most suitable measure for cerebral arterial vessel segmentation from TOF images. We showed that the average Hausdorff distance, especially its balanced version, is best suited for quality assessment of cerebral vessel segmentations. The ranking performance of average distance-based measures was superior in comparison to overlap-based measures, especially in ranking segmentations of good quality. We corroborated the theoretical assumptions that distance-based measures identify more relevant segmentation errors in complex structures like vessel trees due to their consideration of voxel localisation.

Finding a suitable performance measure for a specific segmentation task requires analysing the features of the anatomical structures that are segmented [10]. Cerebral vessel trees have complex boundaries, especially when considering pathologies like the stenosis of a vessel. Cerebral vessel tree segments are remarkably smaller than the background since only around 1% of brain voxels are vessels [8]. Outliers, small false-positive segments far outside of the segment, are also harmful in cerebral vessel segmentation because they often represent false-positive anatomical structures. On theoretical grounds, Taha and colleagues suggested to favour distance-based performance measures for small segments with complex boundaries where outliers are also considered to be important [10]. Our empirical results with bAHD and AHD as the top-performing performance measures confirm these theoretical considerations.

Why average distance-based measures outperformed other measures can be explained by specific measure properties. For example, similarity-based performance measures such as Dice or Sensitivity do not take information about the voxel localisation into consideration. Voxel localisation, however, is of paramount importance in cerebral vessel segmentation. Distance-based performance measures penalise voxels and surfaces that are further away from the ground truth more severely. This allows the distance-based performance measures to recognise a false-positive structure, for example, the superior sagittal sinus, and penalise the error accordingly.

The lack of sensitivity of the Dice coefficient towards specific errors becomes evident when looking at Fig. 4. Here, the severe omission of small vessels leads to a Dice coefficient of 0.960, which is almost identical to that of a minor boundary error of the internal carotid artery with a Dice coefficient of 0.963. bAHD, however, takes voxel localisation into consideration and penalises the severe small vessel error adequately. This shows that the application of measures like the Dice coefficient is problematic. As long as many errors or severe errors are present, both the Dice coefficient and distance-based measures will be sufficient to identify a bad segmentation. When only a few errors are left, i.e. the best segmentation out of a group of good segmentations must be chosen, the Dice coefficient cannot correctly rank the segmentations anymore. The work of Hilbert et al. also corroborates this. They found no significant differences in Dice values when comparing different high-performing architectures but did find significant differences in the average Hausdorff distance values [5].

These considerations have direct implications for the further development of novel vessel segmentation algorithms.

On one hand, research has focused on developing completely new [30], modified [26] or combined [14] performance measures that are more sensitive to errors and have wider score ranges to distinguish between subtle differences between ground truth and segmentation. For example, Chang et al. proposed Conformity instead of DICE and Sensibility instead of Specificity. These two new performance measures promised better performance in recognising errors and detecting minor variabilities in segmentations due to their wider score range [28]. The wider score ranges have also been confirmed in our analysis by the index of dispersion (Table 5). Conformity and Sensibility should thus be preferred over Dice and Specificity, respectively.

On the other hand, our results have direct implications for the training process of deep learning applications. During the training process, the algorithm must be given a mathematical formula according to which it can decide how erroneous the current model’s segmentations are. This error definition, so-called loss function in deep learning terminology is minimised during training and consequently used for model adaptation. Currently, Dice coefficient based loss functions are in widespread use [8, 31–33]. Based on the previous considerations, it is evident that such a loss function will experience a ceiling effect and will not identify the optimal segmentation. Thus, we recommend the utilisation of loss functions based on average Hausdorff distance measures as the default loss function for arterial brain vessel segmentation [34, 35].

Our results also argue against the utilisation of single measures. Simultaneous usage of multiple measures for performance assessment may reveal aspects of the tested segmentations, which may be overlooked by relying solely on one performance measure [36]. In this sense, using an additional distance-based performance measure may reveal contour errors or outliers that may compromise the segmentation quality. The 95% Hausdorff Distance, for example, quantifies the largest error of a segmentation as the longest distance one has to travel from a point in one of the two sets to its closest point in the other set [27]. Thus, the 95% Hausdorff Distance provides a different perspective on the quality of the segmentation at hand. We argue that reporting Dice for comparability and overlap-based evaluation, reporting bAHD for capturing more relevant errors, and reporting 95% Hausdorff distance for quantifying the largest segmentation error is a suitable protocol to assess segmentation quality of cerebral vessel segmentations.

Our study has limitations. First, the predefined visual scoring was only performed by one rater due to the highly time-consuming nature of scoring nearly 3000 segmentations. To mitigate this limitation, we performed a validation analysis of visual scores in a random subset which showed a high similarity of scores assigned by two independent raters. This high similarity in the scoring argues in favor of the robustness of our results. Second, we analysed a large amount of 22 measures, but could not analyse all existing performance measures due to availability constraints in the analysis software. Thus, it cannot be ruled out that other measures might exhibit better performance than the ones identified in the current work. Third, the different types of technically designed errors were not weighted according to their clinical impact on treatment decisions. Fourth, our work was performed in images of 3D-TOF-MRI only. However, it is likely that the results are transferable to other 3D neuroimaging modalities such as computed tomography (CT). Fifth, our study included a limited number of 10 patients. Time intensive manual error creation and subsequent visual scoring are the main limiting factors to increase the number of patients. However, it is important to note that our analysis mainly depends on a large number and the variable selection of different errors and less on the number of patients. This is due to the fact that the variability of changes in the vasculature introduced by the errors is far larger than the anatomical variation between patients.

## Conclusions

Out of all performance measures analysed in this work, average distance based measures are most suited to identify the optimal segmentations for arterial brain vessel segmentation from 3D-TOF-MRI. Our work has the potential to accelerate the validation and development of novel vessel segmentation approaches.

## Supplementary Information

**Additional file 1**. Visualisations of manually created segmentation errors.

**Additional file 2**. Performance measure rankings of individual patients.

## Data Availability

At the current time-point the imaging data cannot be made publicly accessible due to data protection but the authors will make efforts in the future, thus this status might change. The code for the performance measure selection framework is available under the following GitHub repository: https://github.com/prediction2020/arterial_vessel_measures.

## Abbreviations

TOF MRA: Time-of-flight MR-Angiography
ICA: Internal carotid artery
Pcom: Posterior communicating artery
M1: The sphenoidal segment of the middle cerebral artery
IoD: Index of dispersion
PM: Performance measure
