# Supplementary material for: An evaluation of performance measures for arterial brain vessel segmentation
Source: BMC Med Imaging. 2021 Jul 16;21:113. doi: 10.1186/s12880-021-00644-x (PMC8283850; doi:10.1186/s12880-021-00644-x)
Supplement: Supplementary file 1 — Additional file 1. Visualisations of manually created segmentation errors. [file 12880_2021_644_MOESM1_ESM.docx]

**Additional File 1**

**Description:** This file contains all manually created errors that are introduced to the ground truth segmentation of an example patient. Please refer to Table 1 in the publication for an overview of all errors.


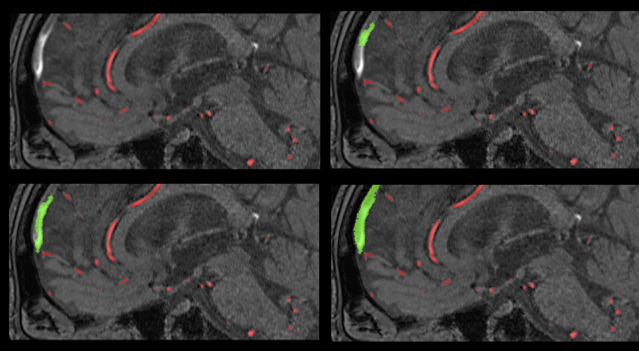


**Error 1: Sinus Sagittalis Superior (SSS) error**

Gradually increasing number of false positive voxels within the SSS were introduced to the ground truth. Sagittal view. Top left: Ground truth, Top right: subtle SSS error, Bottom left: moderate SSS error, Bottom right: severe SSS error. Red: True positive voxels, Green: False positive voxels.


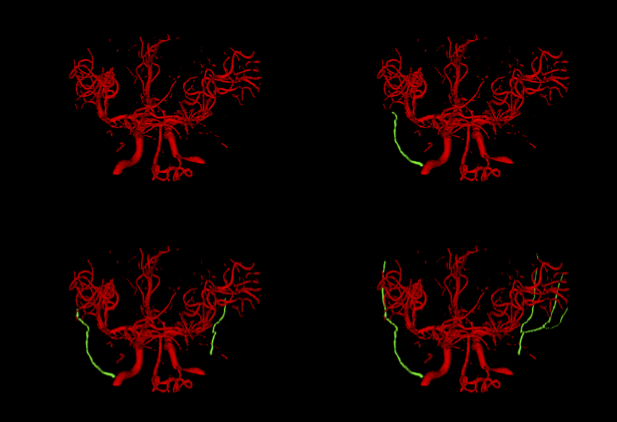


**Error 2: Middle meningeal artery (MMA) error**

Top left: Ground truth, Top right: subtle MMA error, Bottom left: moderate MMA error, Bottom right: severe MMA error. Red: True positive voxels, Green: False positive voxels


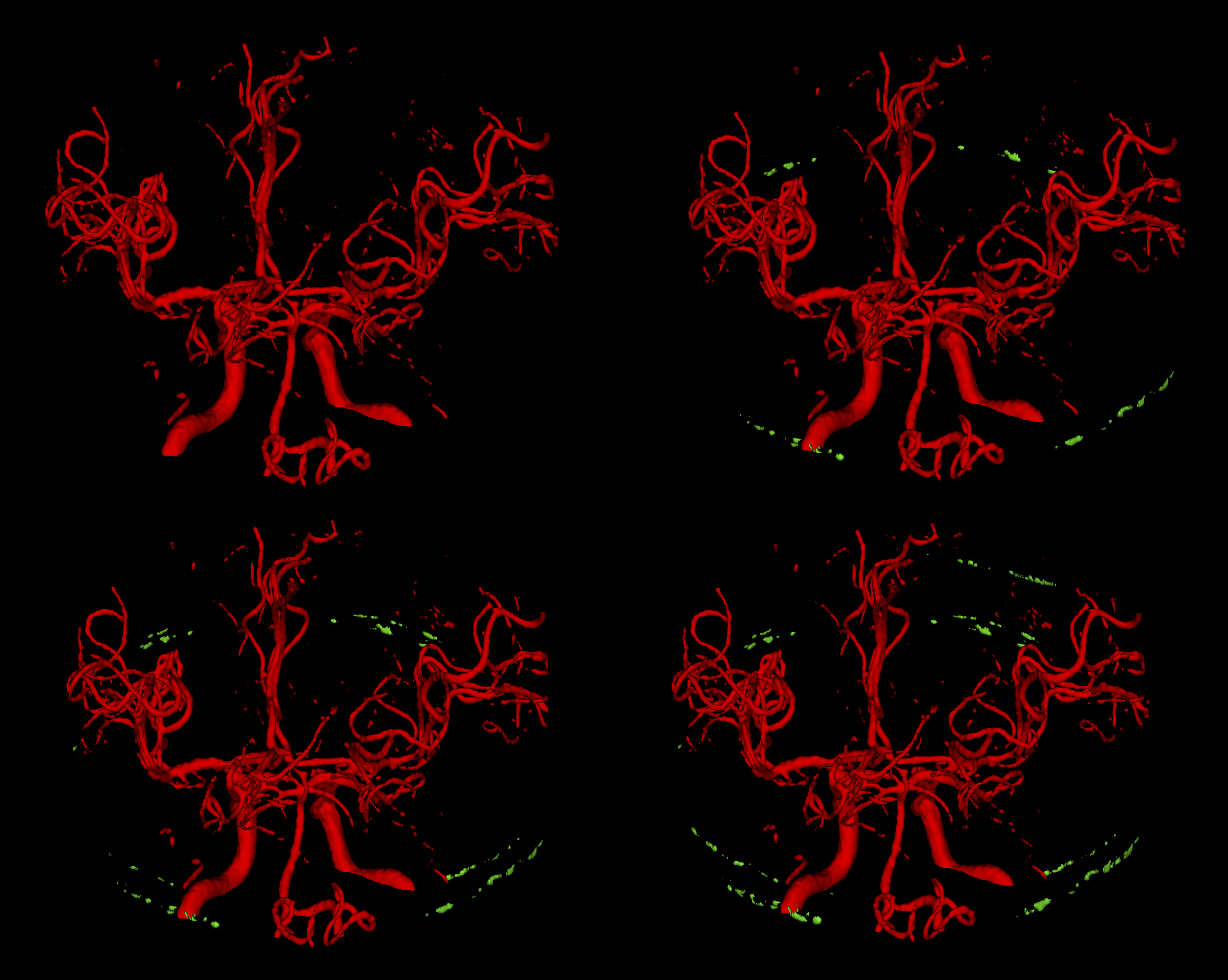


**Error 3: Hyperintense meninges error**

Top left: Ground truth, Top right: subtle hyperintense meninges error, Bottom left: moderate hyperintense meninges error, Bottom right: severe hyperintense meninges error. Red: True positive voxels, Green: False positive voxels


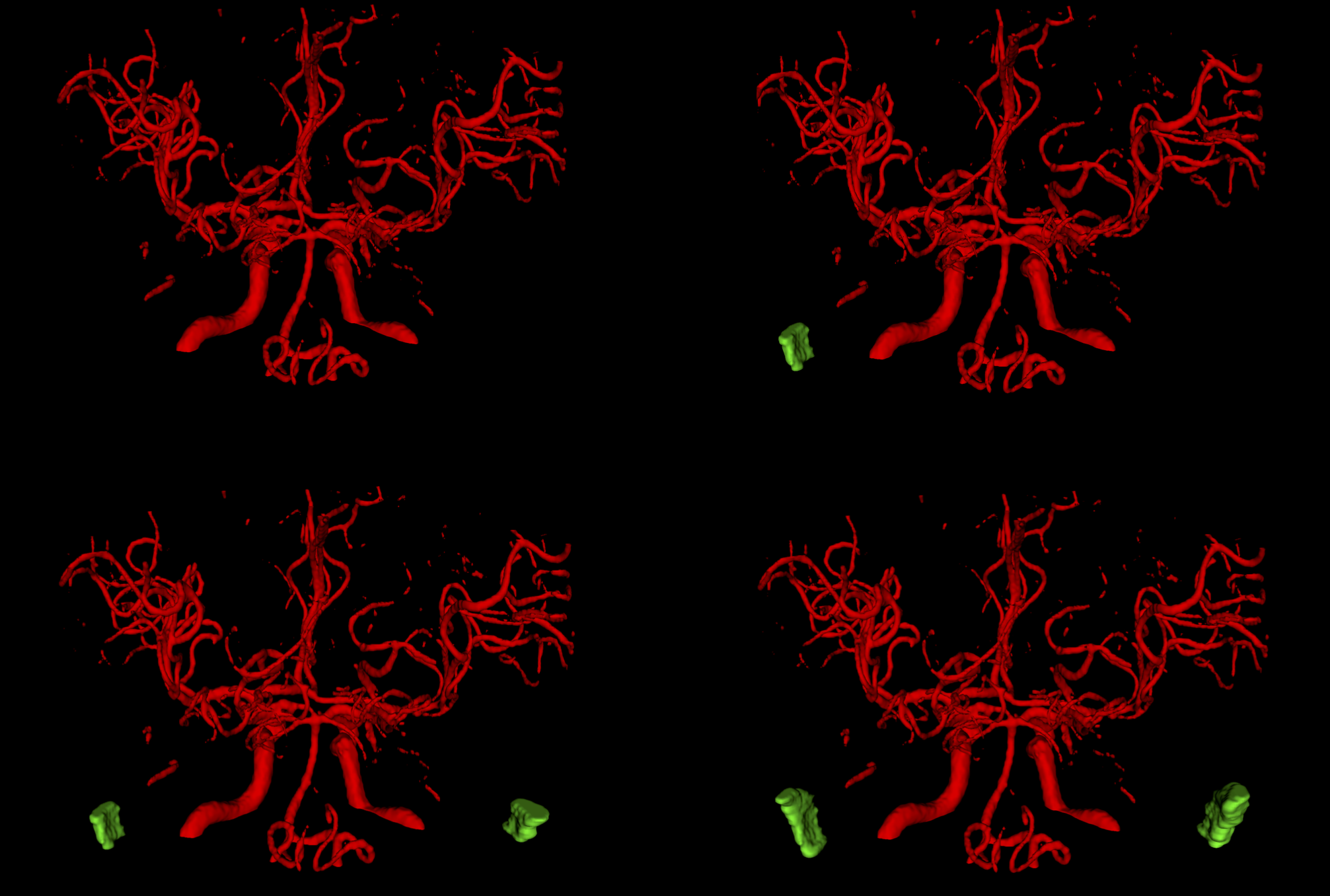


**Error 4: Sigmoid sinus error**

Top left: Ground truth, Top right: subtle sigmoid sinus error, Bottom left: moderate sigmoid sinus error, Bottom right: severe sigmoid sinus error. Red: True positive voxels, Green: False positive voxels


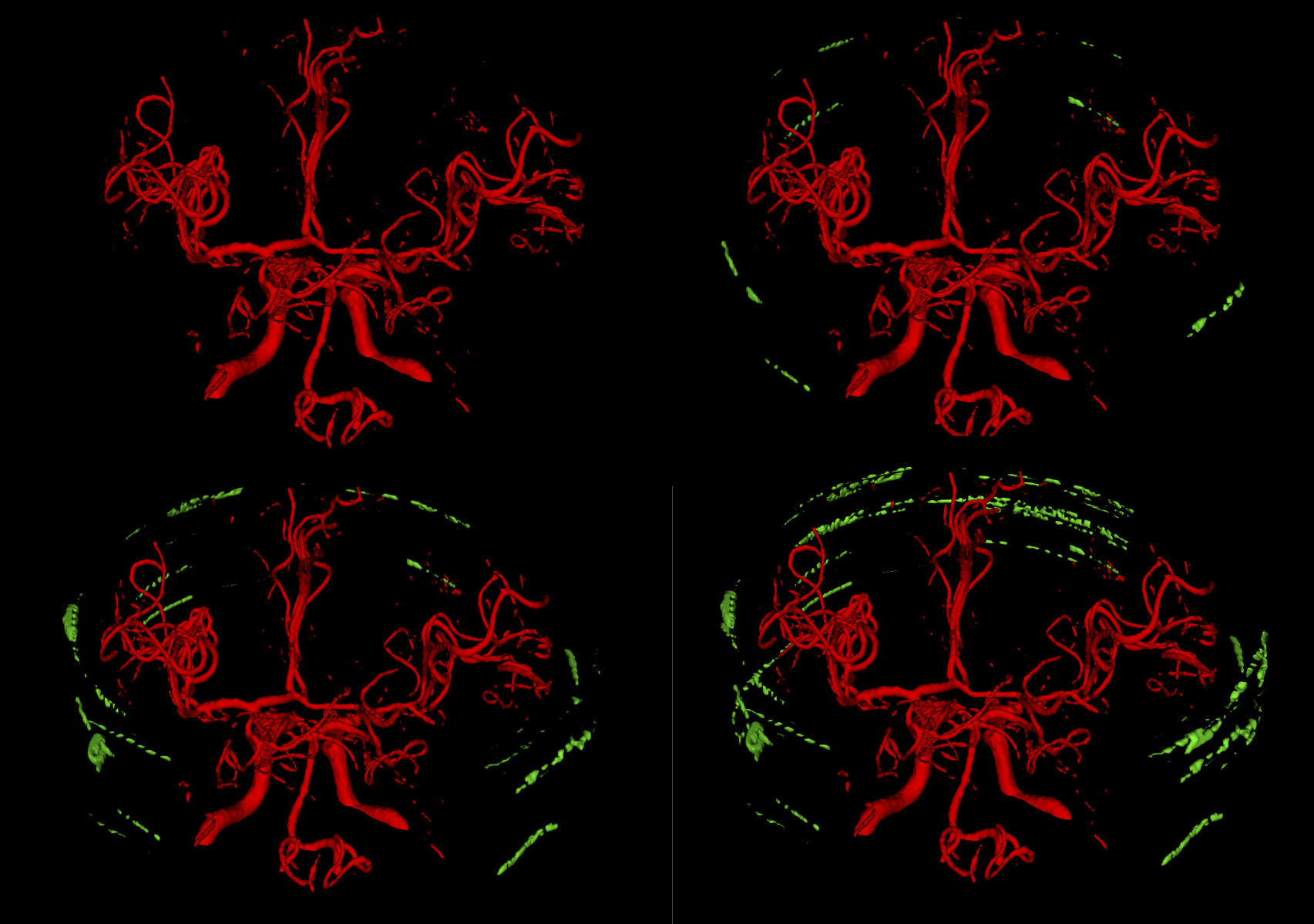


**Error 5: Skull error**

Top left: Ground truth, Top right: subtle skull error, Bottom left: moderate skull error, Bottom right: severe skull error. Red: True positive voxels, Green: False positive voxels


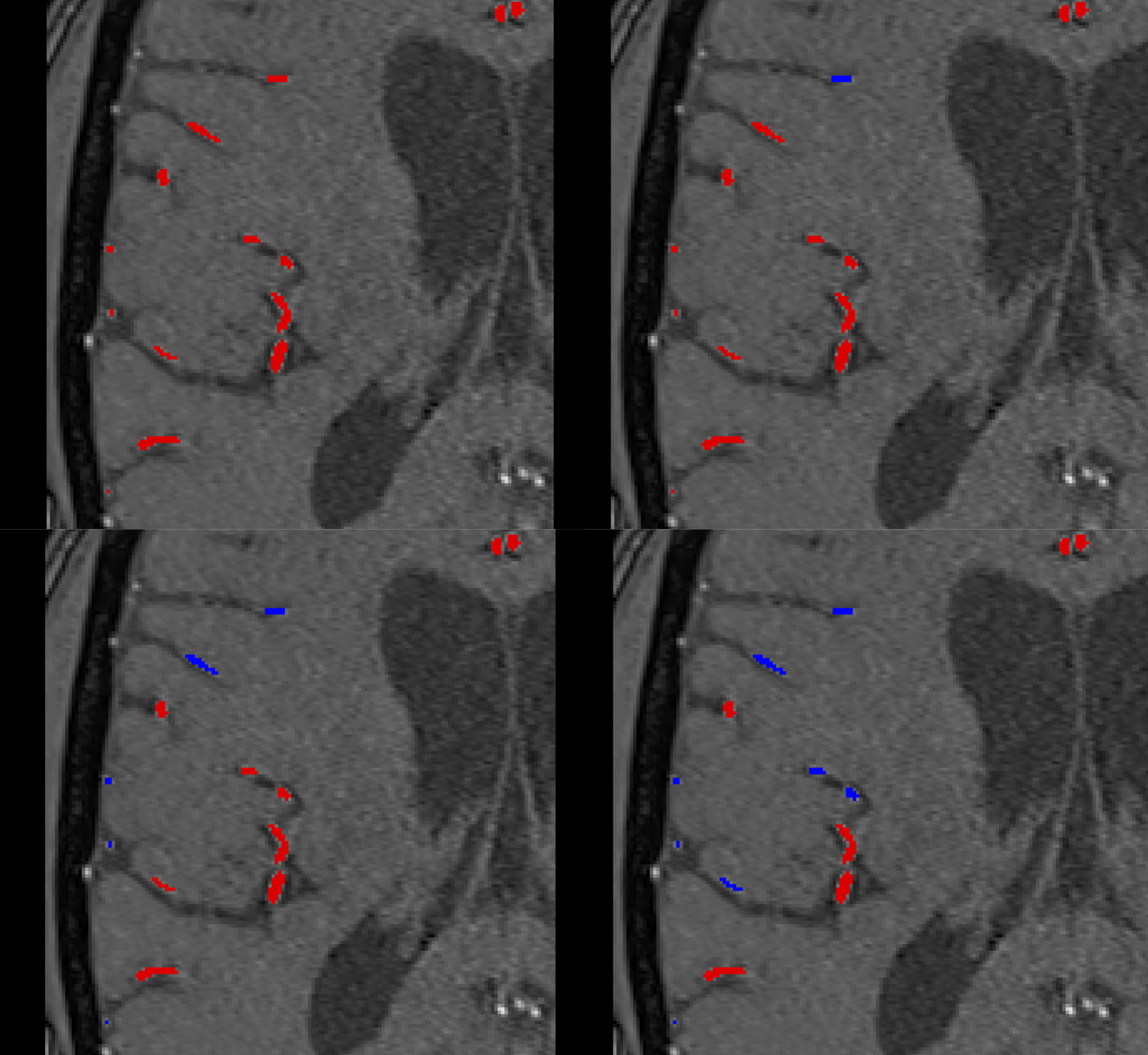


**Error 6: Small vessel error**

Top left: Ground truth, Top right: subtle small vessel error, Bottom left: moderate small vessel error, Bottom right: severe small vessel error. Red: True positive voxels, Blue: False negative voxels


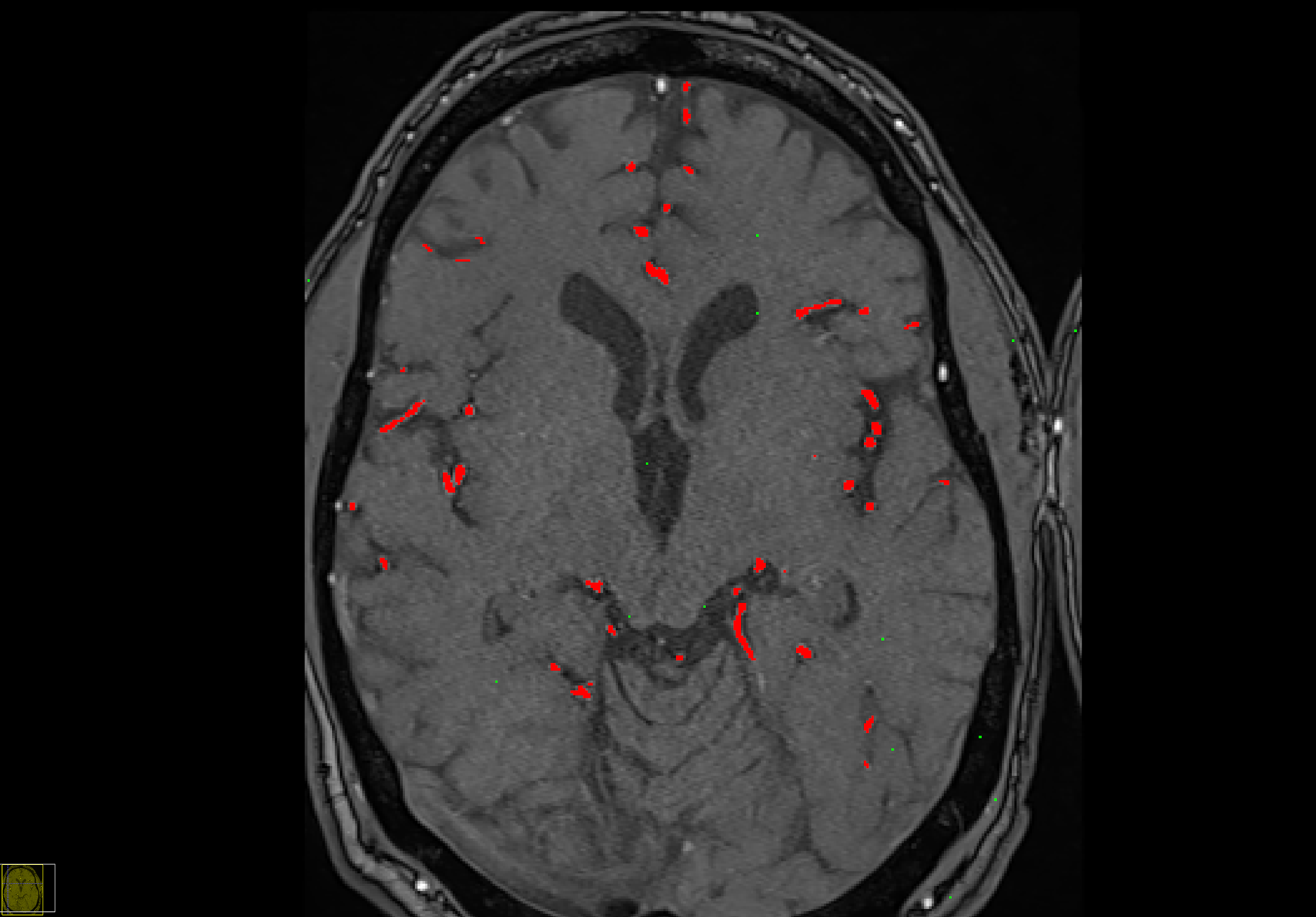


**Error 7: Random voxel error**

Random voxel error selects voxels (Subtle: 1%, Moderate: 2%, Severe: 3% of all ground truth voxels) from the original TOF MRA and adds them randomly scattered to the ground truth image. Red: True positive voxels, Green: False positive voxels


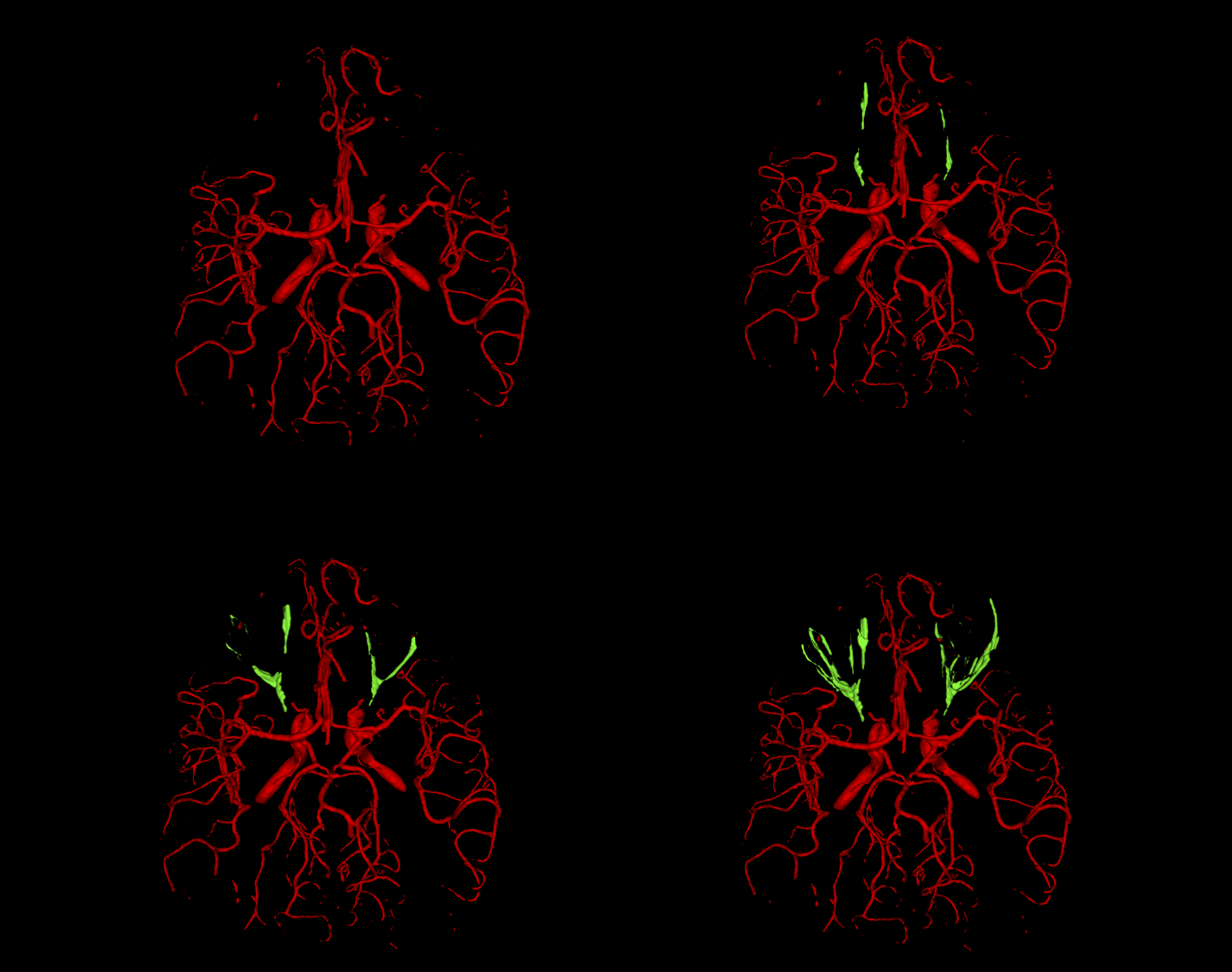


**Error 8: Orbit error**

Top left: Ground truth, Top right: subtle orbit error, Bottom left: moderate orbit error, Bottom right: severe orbit error. Red: True positive voxels, Green: False positive voxels


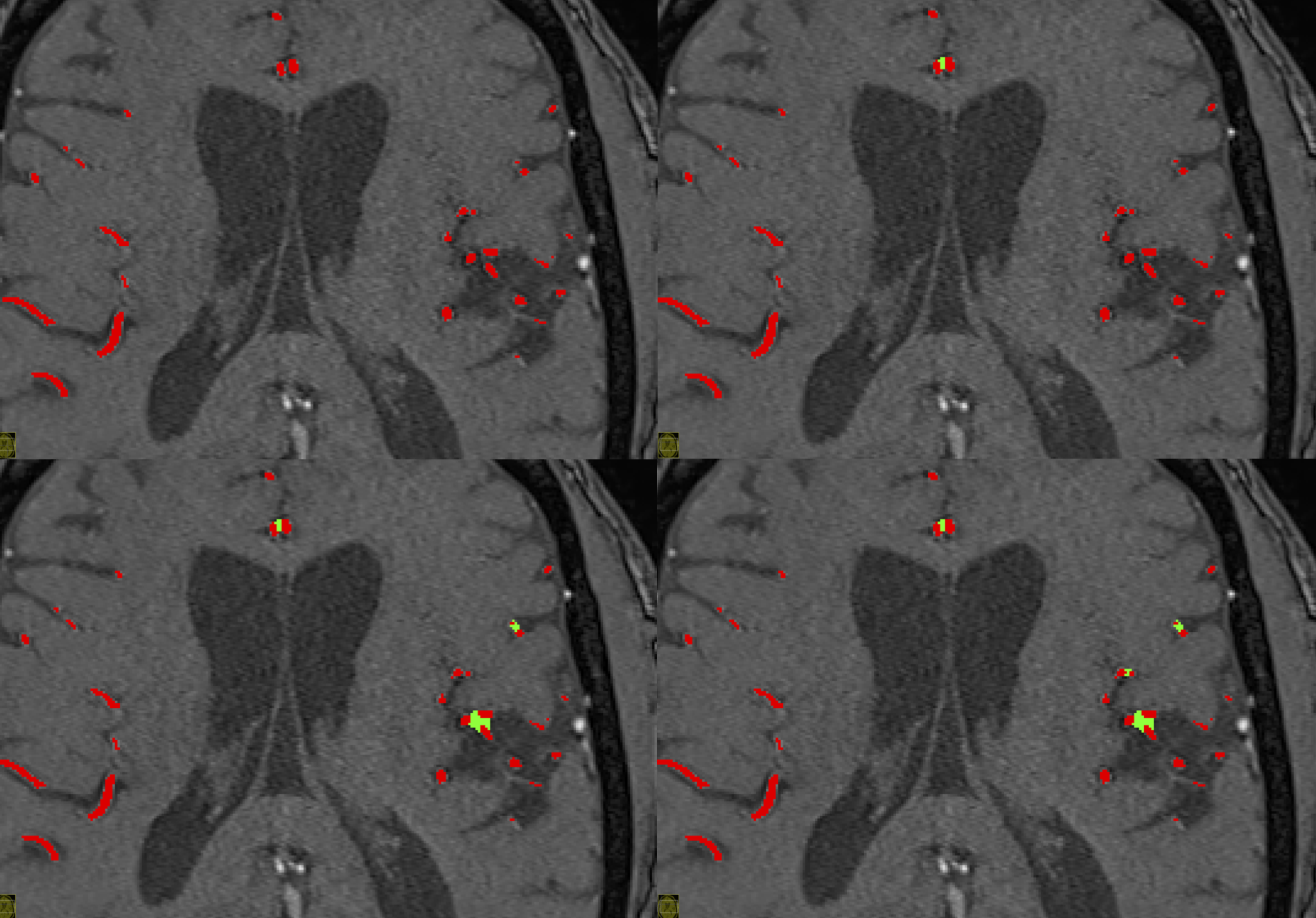


**Error 9: Merge/Separation Error**

Vessels in close proximity to each other were merged by adding false positive voxels near A2 segments or M3-M4 segments. Top left: Ground truth, Top right: subtle merge error, Bottom left: moderate merge error, Bottom right: severe merge error. Red: True positive voxels, Green: False positive voxels


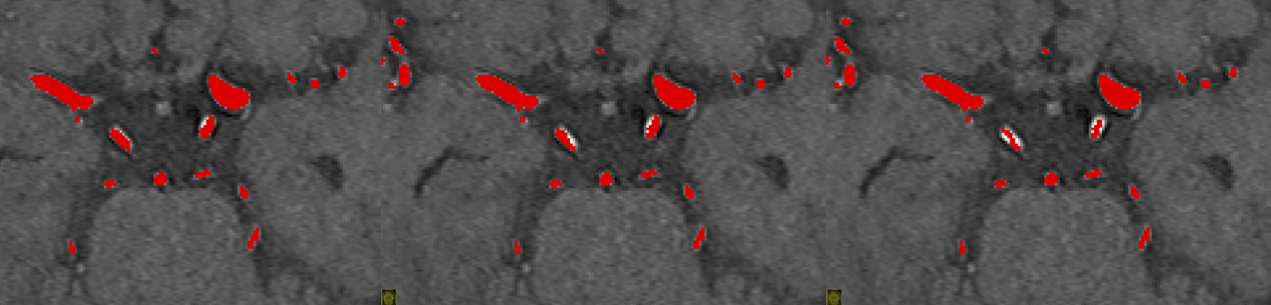


**Error 10: Pcom under-segmentation**

Left: Pcom under-segmentation subtle error, Middle: Pcom under-segmentation moderate error, Right: Pcom under-segmentation severe error, Red: True positive voxels

**Error 11: Pcom missing**
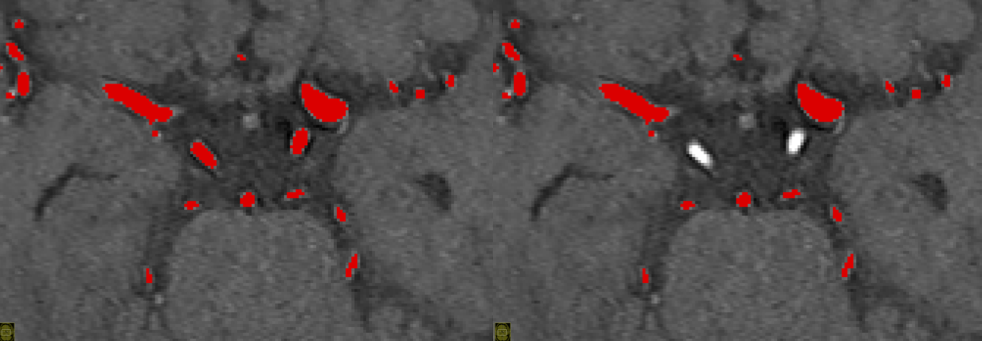


Left: Ground truth, Right: Pcom segment omitted. Red: True positive voxels


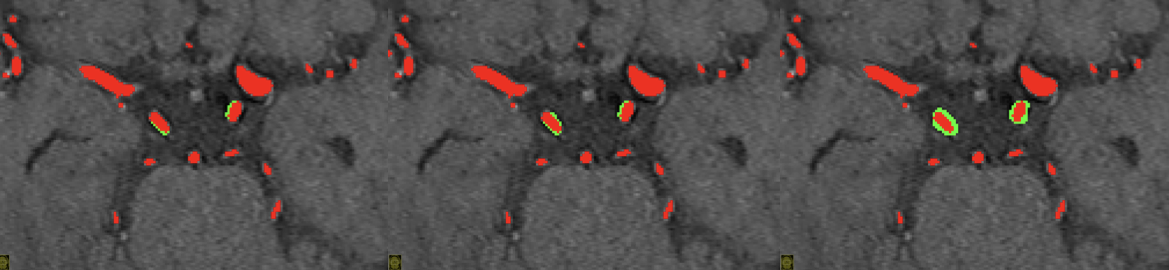
**Error 12: Pcom over-segmentation**

Left: Pcom over-segmentation subtle error, Middle: Pcom over-segmentation moderate error, Right: Pcom over-segmentation severe error. Red: True positive voxels, Green: False positive voxels


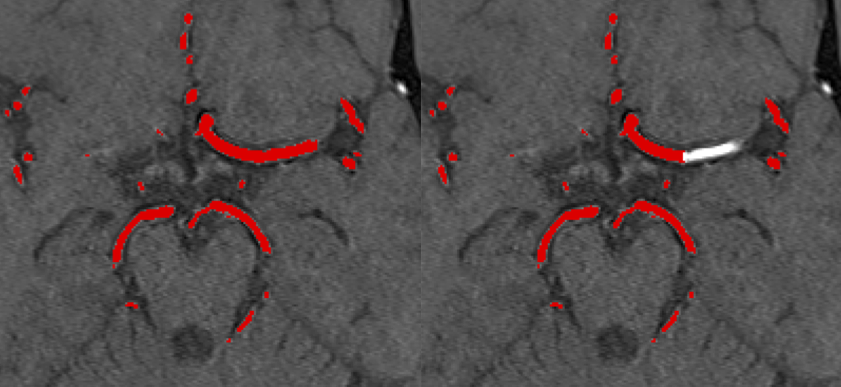


**Error 13: M1 missing**

Left: Ground truth, Right: M1 segment is omitted bilaterally (not shown in this image).

Red: True positive voxels


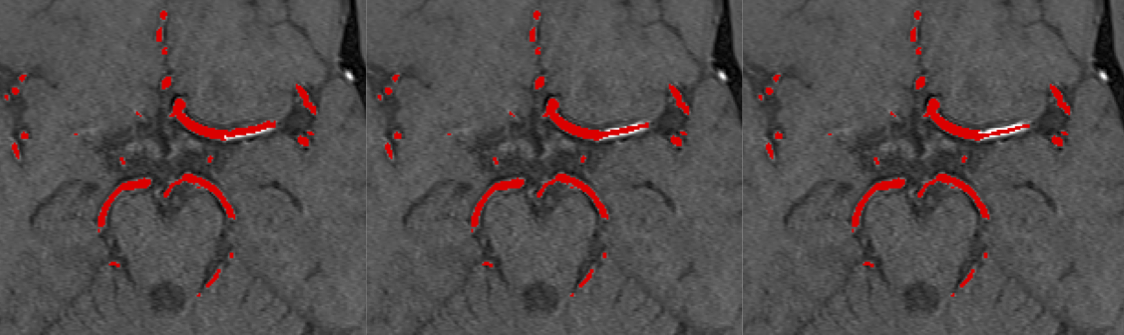


**Error 14: M1 under-segmentation**

Left: M1 under-segmentation subtle error, Middle: M1 under-segmentation moderate error, Right: M1 under-segmentation severe error. Red: True positive voxels

**
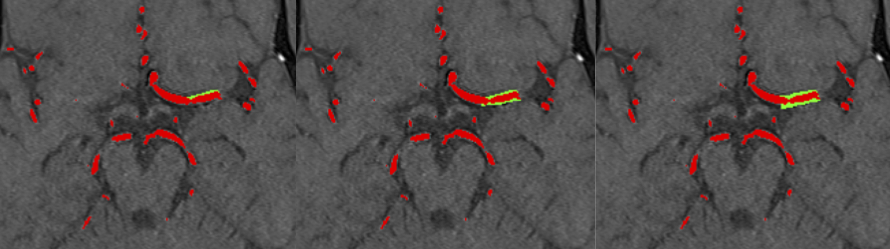
**

**Error 15: M1 over segmentation**

Left: M1 over-segmentation subtle error, Middle: M1 over-segmentation moderate error, Right: M1 over-segmentation severe error. Red: True positive voxels, Green: False positive voxels


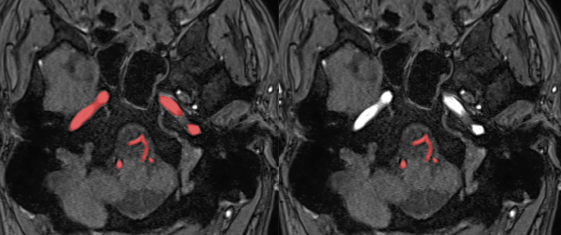


**Error 16: Internal carotid artery(ICA) missing error**

Left: Ground truth, Right: ICA is omitted bilaterally. Red: True positive voxels,

ICA under-segmentation


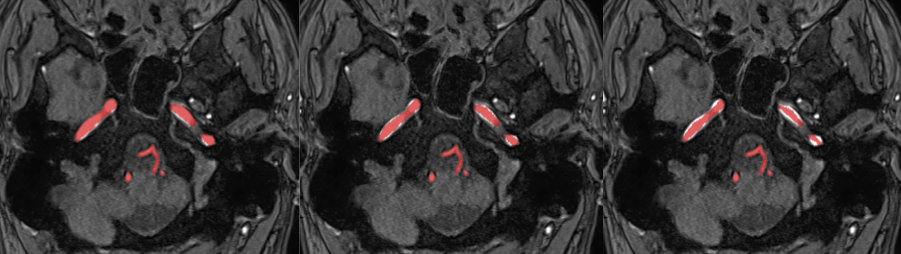


**Error 17: Internal carotid artery (ICA) under-segmentation**

Left: ICA under-segmentation subtle error, Middle: ICA under-segmentation moderate error, Right: ICA under-segmentation severe error. Red: True positive voxels


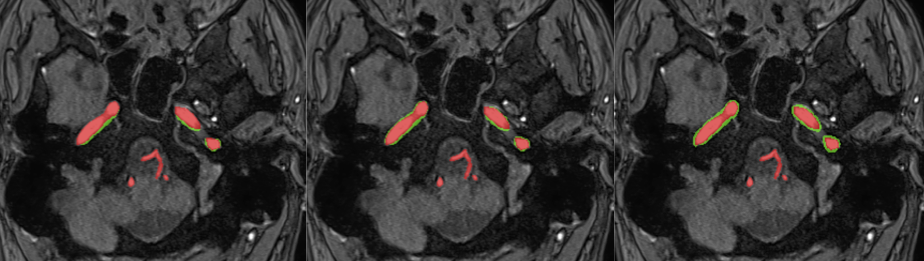


**Error 18: Internal carotid artery (ICA) over-segmentation**

Left: ICA over-segmentation subtle error, Middle: ICA over-segmentation moderate error, Right: ICA over-segmentation severe error. Red: True positive voxels Green: False positive voxels
