# Supplementary material for: An evaluation of performance measures for arterial brain vessel segmentation
Source: BMC Med Imaging. 2021 Jul 16;21:113. doi: 10.1186/s12880-021-00644-x (PMC8283850; doi:10.1186/s12880-021-00644-x)
Supplement: Supplementary file 2 — Additional file 2. Performance measure rankings of individual patients. [file 12880_2021_644_MOESM2_ESM.docx]

**Table: Performance measure rankings for individual patients**

| **Patient 1** | | | **Patient 2** | | | **Patient 3** | | | **Patient 4** | | | **Patient 5** | | |
| --- | --- | --- | --- | --- | --- | --- | --- | --- | --- | --- | --- | --- | --- | --- |
| **PM** | **Rank** | **rho** | **PM** | **Rank** | **rho** | **PM** | **Rank** | **rho** | **PM** | **Rank** | **rho** | **PM** | **Rank** | **rho** |
| ICC | 1 | 0.964 | bAHD | 1 | 0.944 | AHD | 1 | 0.958 | bAHD | 1 | 0.956 | AHD | 1 | 0.963 |
| DICE | 1 | 0.964 | AHD | 2 | 0.941 | bAHD | 2 | 0.957 | AHD | 2 | 0.949 | bAHD | 2 | 0.963 |
| CNF | 1 | 0.964 | KAP | 3 | 0.906 | ACC | 3 | 0.930 | ACC | 3 | 0.927 | RI | 3 | 0.942 |
| JAC | 1 | 0.964 | ARI | 4 | 0.906 | RI | 4 | 0.930 | RI | 4 | 0.927 | ACC | 4 | 0.942 |
| KAP | 5 | 0.964 | PBD | 5 | 0.905 | GCE | 5 | 0.930 | GCE | 5 | 0.925 | GCE | 5 | 0.941 |
| ARI | 6 | 0.964 | DICE | 6 | 0.905 | VOI | 6 | 0.930 | VOI | 6 | 0.924 | VOI | 6 | 0.939 |
| PBD | 7 | 0.964 | ICC | 7 | 0.905 | ARI | 7 | 0.927 | ARI | 7 | 0.915 | ARI | 7 | 0.938 |
| VOI | 8 | 0.958 | CNF | 7 | 0.905 | KAP | 8 | 0.927 | KAP | 8 | 0.915 | KAP | 8 | 0.938 |
| GCE | 9 | 0.958 | JAC | 7 | 0.905 | ICC | 9 | 0.927 | PBD | 9 | 0.915 | ICC | 9 | 0.937 |
| ACC | 10 | 0.958 | VOI | 10 | 0.902 | DICE | 9 | 0.927 | ICC | 10 | 0.915 | DICE | 9 | 0.937 |
| RI | 11 | 0.958 | GCE | 11 | 0.902 | CNF | 9 | 0.927 | DICE | 11 | 0.915 | CNF | 9 | 0.937 |
| AHD | 12 | 0.915 | RI | 12 | 0.897 | JAC | 9 | 0.927 | CNF | 11 | 0.915 | JAC | 9 | 0.937 |
| bAHD | 13 | 0.908 | ACC | 13 | 0.897 | PBD | 13 | 0.927 | JAC | 11 | 0.915 | PBD | 13 | 0.937 |
| MI | 14 | 0.869 | PRC | 14 | 0.853 | PRC | 14 | 0.853 | PRC | 14 | 0.829 | PRC | 14 | 0.868 |
| VS | 15 | 0.849 | SP | 15 | 0.825 | SP | 15 | 0.814 | SB | 15 | 0.780 | SP | 15 | 0.837 |
| PRC | 16 | 0.817 | SB | 16 | 0.825 | SB | 16 | 0.814 | SP | 16 | 0.779 | SB | 16 | 0.837 |
| MHD | 17 | 0.803 | MI | 17 | 0.753 | MI | 17 | 0.762 | MHD | 17 | 0.723 | MHD | 17 | 0.768 |
| SP | 18 | 0.783 | VS | 18 | 0.718 | MHD | 18 | 0.759 | MI | 18 | 0.664 | MI | 18 | 0.761 |
| SB | 19 | 0.782 | MHD | 19 | 0.578 | VS | 19 | 0.674 | VS | 19 | 0.661 | VS | 19 | 0.718 |
| AUC | 20 | 0.402 | HD95 | 20 | 0.425 | HD95 | 20 | 0.432 | HD95 | 20 | 0.456 | AUC | 20 | 0.393 |
| SNS | 21 | 0.331 | AUC | 21 | 0.396 | AUC | 21 | 0.422 | AUC | 21 | 0.325 | HD95 | 21 | 0.368 |
| HD95 | 22 | 0.315 | SNS | 22 | 0.329 | SNS | 22 | 0.372 | SNS | 22 | 0.258 | SNS | 22 | 0.343 |
|  | | | | | | | | | | | | | | |
| **Patient 6** | | | **Patient 7** | | | **Patient 8** | | | **Patient 9** | | | **Patient 10** | | |
| **PM** | **Rank** | **rho** | **PM** | **Rank** | **rho** | **PM** | **Rank** | **rho** | **PM** | **Rank** | **rho** | **PM** | **Rank** | **rho** |
| bAHD | 1 | 0.952 | bAHD | 1 | 0.956 | ARI | 1 | 0.944 | bAHD | 1 | 0.962 | bAHD | 1 | 0.959 |
| AHD | 2 | 0.949 | AHD | 2 | 0.951 | PBD | 2 | 0.943 | AHD | 2 | 0.956 | AHD | 2 | 0.955 |
| GCE | 3 | 0.936 | GCE | 3 | 0.938 | ICC | 3 | 0.943 | RI | 3 | 0.916 | ACC | 3 | 0.941 |
| VOI | 4 | 0.935 | VOI | 4 | 0.938 | DICE | 3 | 0.943 | ACC | 4 | 0.916 | RI | 4 | 0.940 |
| RI | 5 | 0.935 | RI | 5 | 0.938 | CNF | 3 | 0.943 | VOI | 5 | 0.916 | GCE | 5 | 0.940 |
| ACC | 6 | 0.935 | ACC | 6 | 0.938 | JAC | 3 | 0.943 | GCE | 6 | 0.916 | VOI | 6 | 0.940 |
| ARI | 7 | 0.929 | ARI | 7 | 0.936 | KAP | 7 | 0.943 | ARI | 7 | 0.898 | ARI | 7 | 0.939 |
| KAP | 8 | 0.929 | KAP | 8 | 0.936 | AHD | 8 | 0.939 | KAP | 8 | 0.897 | KAP | 8 | 0.939 |
| ICC | 9 | 0.929 | PBD | 9 | 0.935 | bAHD | 9 | 0.938 | ICC | 9 | 0.897 | DICE | 9 | 0.939 |
| DICE | 9 | 0.929 | ICC | 10 | 0.935 | RI | 10 | 0.937 | DICE | 9 | 0.897 | JAC | 9 | 0.939 |
| CNF | 9 | 0.929 | DICE | 10 | 0.935 | ACC | 11 | 0.937 | CNF | 9 | 0.897 | ICC | 9 | 0.939 |
| JAC | 9 | 0.929 | CNF | 10 | 0.935 | VOI | 12 | 0.937 | JAC | 9 | 0.897 | CNF | 9 | 0.939 |
| PBD | 13 | 0.929 | JAC | 10 | 0.935 | GCE | 13 | 0.937 | PBD | 13 | 0.897 | PBD | 13 | 0.939 |
| PRC | 14 | 0.868 | PRC | 14 | 0.875 | PRC | 14 | 0.838 | PRC | 14 | 0.863 | PRC | 14 | 0.880 |
| SP | 15 | 0.830 | SP | 15 | 0.841 | SP | 15 | 0.798 | SP | 15 | 0.803 | SB | 15 | 0.833 |
| SB | 16 | 0.830 | SB | 16 | 0.841 | SB | 16 | 0.798 | SB | 16 | 0.803 | SP | 16 | 0.833 |
| VS | 17 | 0.726 | VS | 17 | 0.796 | VS | 17 | 0.765 | MHD | 17 | 0.732 | VS | 17 | 0.801 |
| MI | 18 | 0.691 | MI | 18 | 0.742 | MI | 18 | 0.761 | MI | 18 | 0.716 | MHD | 18 | 0.776 |
| MHD | 19 | 0.672 | MHD | 19 | 0.530 | MHD | 19 | 0.629 | VS | 19 | 0.703 | MI | 19 | 0.756 |
| HD95 | 20 | 0.404 | HD95 | 20 | 0.421 | HD95 | 20 | 0.381 | HD95 | 20 | 0.416 | HD95 | 20 | 0.436 |
| AUC | 21 | 0.307 | AUC | 21 | 0.376 | AUC | 21 | 0.379 | AUC | 21 | 0.362 | AUC | 21 | 0.300 |
| SNS | 22 | 0.237 | SNS | 22 | 0.310 | SNS | 22 | 0.317 | SNS | 22 | 0.299 | SNS | 22 | 0.226 |

The correlation of visual scores and performance measure rankings are given for each of the patients. In 8 out of the 10 tested patients an average distance based performance measure, either the balanced average Hausdorff distance or the classic average Hausdorff distance, led the rankings.

Please note that the results are rounded after the third decimal point but exact values were used to calculate the ranks in the Rank column. This leads to different ranks of performance measures although the reported spearman correlation coefficients are the same. (Patient 1 see JAC and KAP). These small differences in the spearman correlation coefficients are however not relevant regarding performance measure characteristics. *PM: Performance measure rho: Spearman correlation coefficient.*
